# Effects of *Euglena gracilis* EOD-1 Ingestion on Salivary IgA Reactivity and Health-Related Quality of Life in Humans

**DOI:** 10.3390/nu11051144

**Published:** 2019-05-22

**Authors:** Ken-ichi Ishibashi, Machiko Nishioka, Nobuteru Onaka, Madoka Takahashi, Daisuke Yamanaka, Yoshiyuki Adachi, Naohito Ohno

**Affiliations:** 1 Laboratory for Immunopharmacology of Microbial Products, School of Pharmacy, Tokyo University of Pharmacy and Life Sciences, Hachioji, Tokyo 192-0392, Japan; ymnkd@toyaku.ac.jp (D.Y.); adachiyo@toyaku.ac.jp (Y.A.); ohnonao@toyaku.ac.jp (N.O.); 2 Kobelco Eco-Solutions Co., Ltd., Kobe, Hyogo 651-2241, Japan; m.nishioka@kobelco-eco.co.jp (M.N.); n.onaka@kobelco-eco.co.jp (N.O.); md.takahashi@kobelco-eco.co.jp (M.T.)

**Keywords:** paramylon, β-1, 3-glucan, antibody, IgA, QOL

## Abstract

*Euglena gracilis* EOD-1, a microalgal strain known for high yields of the β-1, 3-glucan paramylon, is suggested to function as a dietary fiber and enhance immunity. Here, we aimed to investigate the effects of *E. gracilis* EOD-1 biomass (EOD1BM) ingestion on immunoglobulin A (IgA) antibody titers in saliva, its reactivity, and the health-related quality of life (QOL) in humans. Reacting human immunoglobulin preparations and saliva with paramylon granules revealed the presence of anti-paramylon antibodies in the blood and saliva. We conducted a placebo-controlled, double-blind, crossover study involving 13 healthy subjects who ingested the placebo or EOD1BM for 4 weeks. Saliva was collected from each subject before and after ingestion, and IgA titers and *E. gracilis* EOD-1 paramylon (EOD1PM) reactivity were compared. In the EOD1BM Ingestion group, the anti-EOD1PM IgA content and titer increased after EOD1BM ingestion. No such change was observed in the Placebo group. Furthermore, the health-related QOL, especially mental health, increased in the EOD1BM Ingestion group. Thus, EOD1BM ingestion led to the production of paramylon (PM)-specific IgA antibody and increased salivary IgA antibody titers. We demonstrate that EOD1BM ingestion enhanced the immunity in the mucosal surface, evoked an antigen-specific response, and increased the health-related QOL, thereby contributing to health improvement.

## 1. Introduction

*Euglena gracilis*, a type of microalgae, contains several nutrients and is well known for the synthesis of paramylon, a polysaccharide component. Therefore, its functionality as a food ingredient has garnered much attention in recent years, and the immunomodulatory effects of *E. gracilis* in mice and other animals have been reported [1,2,3].

The *E. gracilis* EOD-1 strain can produce high paramylon yields (70–80%) depending on culture conditions [4]. The potential of oral administration of *E. gracilis* EOD-1 biomass (EOD1BM) and purified paramylon (EOD1PM) in improving impaired glucose tolerance and serum lipids has been reported in diet-induced obesity mouse models [4]. Therefore, EOD1BM has been suggested to function as a dietary fiber, and studies exploiting its functionality in improving immunity have been suggested. However, the effect of *E. gracilis* ingestion on the human immune system remains unknown.

Paramylon is a polysaccharide generated as a characteristic cellular reserve material in *Euglena* spp. and is stored as granules. Structurally, it is a linear β-1, 3-glucan arranged in a triple helix; these helices accumulate to form crystalline granules. Paramylon has been reported to exhibit immunomodulatory and anti-allergy effects in animal models [5,6]. Moreover, β-glucans have been reported to show immunomodulatory and antitumor effects and induce cytokine production [7]. Furthermore, β-glucan is found abundantly in nature, such as in fungi and algae, and it is known that the structural and physical properties of β-glucans differ with the source organism. The biological activity of β-glucan is also known to vary with its structural and physical properties [8].

Studies investigating the host recognition mechanism for β-glucans reported dectin-1 as a β-glucan receptor involved in the mediation of β-glucan biological activity [9,10]. Furthermore, we previously reported the occurrence of immunoglobulin (Ig)G, IgM, and IgA anti-β-glucan antibodies as host recognition molecules for β-glucan in both human and animal sera [11,12,13]. The immune complex formed by the binding of β-glucan and its antibody is considered to evoke an antigen-specific immunological response.

The biological activity of *E. gracilis* has been reported in animal models, but its biological activity in humans remains unclear [4,5,6]. Therefore, we hypothesize that EOD1BM, which is rich in paramylon (β-1, 3-glucan), shows immunological activities in humans as well. In this study, we focused on the immunity-enhancing function of EOD1PM in humans. We investigated the reactivity of human serum and salivary anti-EOD1PM antibody and studied the changes in its titer after EOD-1 ingestion. In addition, lifestyle aspects such as eating habits affect the body and mind and may influence the quality of life (QOL) of an individual. It has been reported that β-glucan affects the immunological response and QOL in cancer patients, and that yeast β-glucan improves the mood in stressed human volunteers [14,15]. Therefore, EOD1BM may also affect QOL in human volunteers. Health-related QOL is comprised of the basic elements concerning physical, mental, social life-related, and role-limiting functions. Therefore, we examined the general effect of *E. gracilis* EOD-1 ingestion on the overall health-related QOL of study subjects using the Medical Outcomes Study 36-Item Short Form version 2 (SF-36v2) health survey, a scale that measures health-related QOL comprehensively. This study demonstrates that ingestion of EOD1BM, the microalgal biomass containing β-1, 3-glucan, activated the immune response in the mucosal surface and improved the health-related QOL, especially mental health, in humans. These results highlight the novel effects of *E. gracilis* EOD-1 and suggest its potential as a functional food for humans.

## 2. Materials and Methods

### 2.1. Materials

EOD1BM and EOD1PM were obtained from Kobelco Eco-Solutions Co., Ltd. (Kobe, Japan). Solubilized EOD1 paramylon (sEOD1PM) was dissolved in sodium hydroxide and diluted with 0.1 M carbonate buffer (pH 9.6). Solubilized cell wall glucans from *Candida albicans* and *Aspergillus niger* (CSBG and ASBG, respectively) were prepared using the NaClO oxidation method as described previously [16,17]. Polysaccharide fractions (AgHWE) from *Agaricus brasiliensis* (*Agaricus blazei* Murrill sensu Heinem) were also prepared as described previously [18]. Ovalbumin (OVA) and Dextran T200 were purchased from Sigma-Aldrich (Saint Louis, MO, USA) and Pharmacia (Uppsala, Sweden), respectively.

### 2.2. Ingestion Study

#### 2.2.1. Target Population for Analysis

This study was conducted from September to December 2017. Selected subjects were healthy (not undergoing any treatment for ailments), non-smoking men between the ages of 30 and 70 years. The purpose and content of the study were explained thoroughly via written and oral explanation, and 13 individuals who agreed to participate and provided a written informed consent were enrolled as our target population for analysis. The sample size was chosen based on previous studies of β-glucans in humans [19,20,21]. Subjects who were deemed not suitable for consideration in the ingestion test, such as those who were unable to take measurements at an appropriate time, were excluded, leaving a total of seven subjects for analysis. One of the subjects was excluded because of a BMI over 30. Five subjects were excluded because they took the test and appeared for sample collection at times different from those for the baseline measurements. This test was approved by the Kenshokai Ethics Review Committee (Approval Number: 20170915-1, Approval Date: 15 September 2017).

#### 2.2.2. Test Method

**Figure 1:**
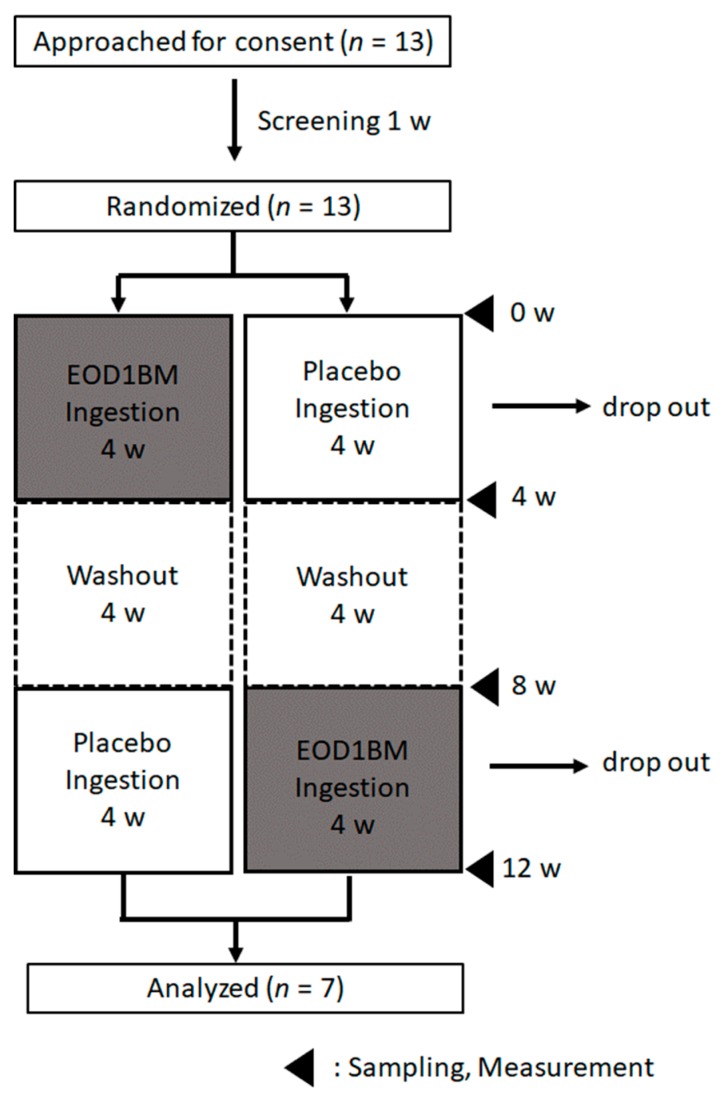
Test design. w: weeks. EOD1BM: *Euglena gracilis* EOD-1 biomass.

The test was designed and performed as a double-blind crossover study (Figure 1). The baseline was measured, and a pre-ingestion examination at 0 weeks (0 w) was performed wherein saliva samples were collected and the subjects were administered several tests and a questionnaire-based survey regarding their feelings associated with the usage. After initial examination, subjects were given the test food (EOD1BM) or a placebo (cornstarch) with an ingestion amount of 500 mg/day and were requested to continue ingesting for a period of 4 weeks. After 4 weeks of ingestion (4 w), they were again subjected to the same tests and questionnaire used at 0 w. Next, a 4-week period of non-ingestion (washout period) was instituted, and the examinations were repeated (8 w). Subjects were then provided with a test food different from that received at 0 w and were requested to ingest it for 4 weeks. Subjects were once again tested after the lapse of these 4 weeks (12 w).

#### 2.2.3. Saliva Collection

Saliva was collected from subjects before the meal and before caffeine intake at a fixed time in the morning in consideration of its daily fluctuation. Saliva samples were collected for 1 min using Salivette (Sarstedt K.K., Tokyo, Japan). The collected saliva samples were stored at −20 °C until further use.

#### 2.2.4. Health-Related QOL Scale Measurement

We used the SF-36v2 [22,23,24] health survey, which is a QOL questionnaire based on universally applicable health-related concepts. For each of the 36 questions, a five-point scale was used for the replies, and the test was analyzed based on the following eight multi-item dimensions of health: physical functioning (PF), role limitations owing to physical problems (RP), body pain (BP), general health perception (GH), energy/vitality (VT), social functioning (SF), role limitations owing to emotional problems (RE), and mental health (MH). Next, the physical, mental, and social aspects of the component summary scores were calculated.

### 2.3. Flow Cytometry Analysis for the Reactivity of Anti-EOD1PM Antibody

EOD1PM suspension was blocked with 0.5% sodium caseinate in phosphate-buffered saline containing 0.05% Tween-20 (PBST pH 7.4; FUJIFILM Wako Pure Chemical, Tokyo, Japan) at 4 °C for 60 min. After centrifugation and removal of supernatants, EOD1PM was reacted with a human immunoglobulin preparation, polyglobin N (Bayer, Berlin, Germany), or pooled saliva at 4 °C for 60 min. The reacted EOD1PM granules were washed with fluorescence activated cell sorter (FACS) buffer (2% calf serum, 0.1% sodium azide, PBS) and incubated with fluorescein isothiocyanate (FITC)-conjugated anti-human IgG or biotin-conjugated anti-human IgA and FITC-conjugated streptavidin (1:200; BioLegend, Santiago, CA, USA) at 4 °C for 60 min and analyzed using a flow cytometer (FACSCalibur, Becton Dickinson, Franklin Lakes, NJ, USA). The histogram shows count data of 1 × 10^4^ EOD1PM.

Next, the specificity of the anti-EOD1PM antibody was tested. EOD1PM particles were blocked with 0.5% sodium caseinate in PBST. Polyglobin N or pooled human saliva were mixed with 100 µg/mL competitive soluble antigen, namely sEOD1PM, CSBG, or OVA. The mixture was incubated at 4 °C for 60 min. The reacted EOD1PM granules were washed with FACS buffer and incubated with FITC-conjugated anti-human IgG or biotin-conjugated anti-human IgA and FITC-conjugated streptavidin (1:200; BioLegend) at 4 °C for 60 min and analyzed using a flow cytometer. The histogram shows count data of 1 × 10^4^ EOD1PM.

### 2.4. ELISA for Anti-B Glucan Antibody in Serum and Saliva

A 96-well microplate was coated with sEOD1PM (25 μg/mL) in 0.1 M carbonate buffer (pH 9.6) by incubation at 4 °C overnight. The plate was washed with PBST and blocked with 0.5% sodium caseinate in PBST at 37 °C for 60 min. The plate was washed with PBST and incubated with human sera (Sigma-Aldrich) or saliva at 37 °C for 60 min. The plate was then washed with PBST and incubated with peroxidase-conjugated anti-human IgG, IgM, or IgA (1:2000; Sigma-Aldrich) in PBST containing 0.5% sodium caseinate and developed with a TMB substrate system (KPL, Gaithersburg, MD, USA). Color development was stopped with 1 N phosphoric acid, and optical density was measured at 450 nm with the absorbance reader MTP-450 (CORONA Electric, Ibaraki, Japan).

For testing the specificity of the anti-sEOD1PM antibody, an ELISA plate was coated with sEOD1PM (25 µg/mL in carbonate buffer) and blocked with 0.5% sodium caseinate in PBST before use. Human sera or pooled human saliva were mixed with serially diluted competitive soluble antigen, namely sEOD1PM, CSBG, OVA, AgHWE, ASBG, or dextran, and then applied to the ELISA plate. The amount of plate-bound Ig was determined using peroxidase-conjugated anti-human IgG or IgA (1:2000).

### 2.5. Statistical Analyses

The Mann–Whitney test was used for comparisons of results from the placebo and EOD1BM groups. The Wilcoxon signed-rank test was used for comparisons of results obtained before and after ingestion. These statistical analyses were performed using MedCalc statistical software version 11.6.1.0 (Acacialaan, Ostend, Belgium).

## 3. Results

### 3.1. Binding of Human Serum and Saliva Immunoglobulin to Paramylon Granules

**Figure 2:**
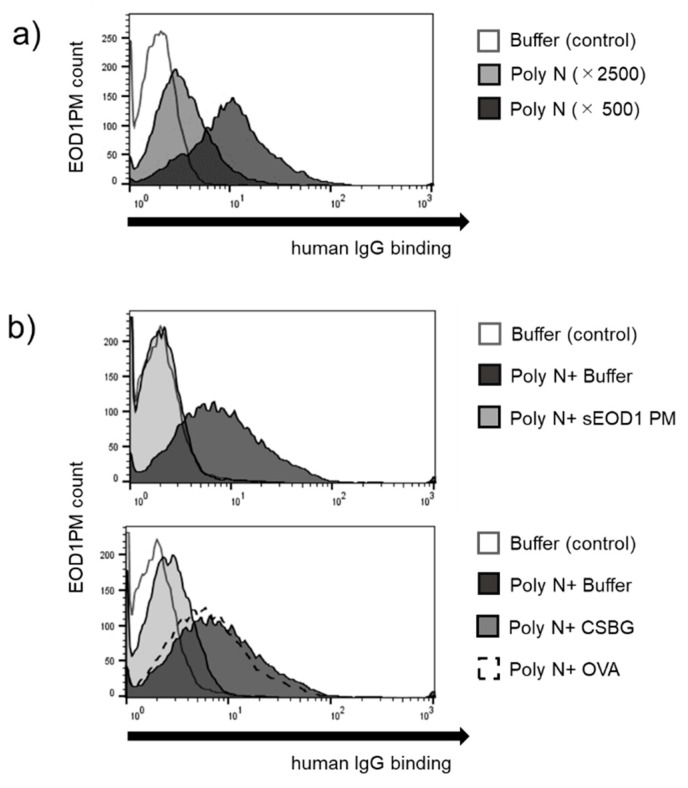
Binding of human immunoglobulin to *Euglena gracilis* EOD-1 paramylon (EOD1PM). (**a**) Reactivity of the anti-EOD1PM antibody. EOD1PM particles were blocked, reacted with polyglobin N, and analyzed by flow cytometry; (**b**) Specificity of the anti-EOD1PM antibody. EOD1PM particles were blocked, reacted with mixture of polyglobin N and competitive soluble antigen, and analyzed using flow cytometry. CSBG: solubilized cell wall glucan from *Candida albicans*; OVA: ovalbumin; sEOD1PM: solubilized EOD1 paramylon.

First, to investigate the binding of human immunoglobulins with paramylon granules, we incubated the granules with immunoglobulin preparations from human sera and analyzed their binding using flow cytometry. Immunoglobulin addition resulted in a concentration-dependent shift in the peak, revealing the binding of human serum immunoglobulin to EOD1PM (Figure 2a).

Next, to examine the specificity of this anti-EOD1PM antibody, sEOD1PM, CSBG, or OVA were mixed with the immunoglobulin preparation, and the competitive binding of EOD1PM granules and solubilized antigen to the immunoglobulin was investigated. sEOD1PM and CSBG addition both suppressed the peak shift and antibody binding; however, OVA addition did not result in such suppression (Figure 2b). These results indicate that the anti-EOD1PM antibody is β-glucan–specific, and cross-reacts with the *Candida*-derived β-glucan.

**Figure 3:**
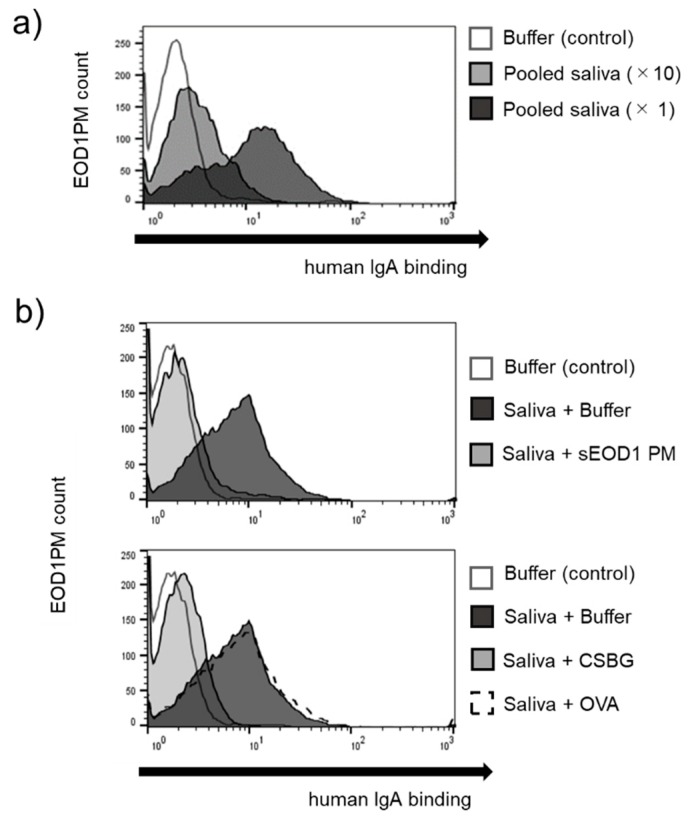
Binding of human saliva to *Euglena gracilis* EOD-1 paramylon (EOD1PM). (**a**) Reactivity of salivary anti-EOD1PM immunoglobulin A (IgA) antibody. EOD1PM particles were blocked, reacted with pooled human saliva, and analyzed by flow cytometry. (**b**) Specificity of salivary anti-EOD1PM IgA antibody. EOD1PM particles were blocked, reacted with a mixture of pooled human saliva and 100 µg/mL competitive soluble antigen, and analyzed using flow cytometry.

To understand the binding of a mucosal antibody secreted in response to exposure of the mucosal surface to foreign antigens, we investigated the anti-EOD1PM IgA antibody in the saliva samples obtained from the study subjects. The addition of saliva shifted the peak in a concentration-dependent matter, indicating the presence of anti-EOD1PM IgA antibody in the saliva (Figure 3). Furthermore, antibody specificity experiments based on competitive reactions with the addition of solubilized antigens revealed that the addition of these antigens suppressed the binding; these results are consistent with those obtained for the serum antibody. These findings indicate that the anti-EOD1PM IgA antibody in the saliva is also β-glucan-specific and cross-reacts with *Candida*-derived β-glucan.

### 3.2. Anti-EOD1PM Antibody Reactivity to Solubilized EOD1PM in ELISA

**Figure 4:**
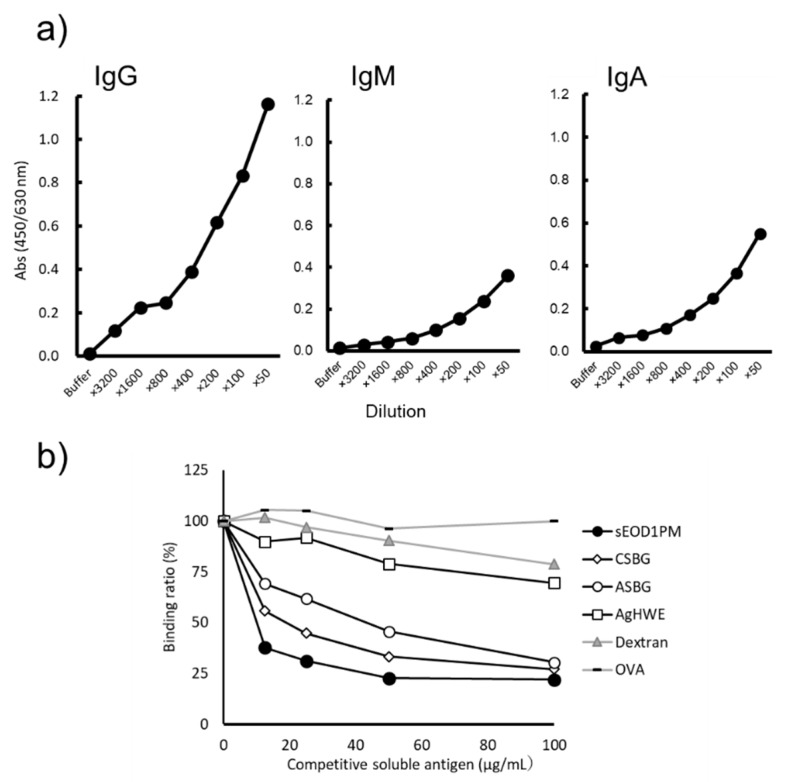
Class of antibody against *Euglena gracilis* EOD-1 paramylon (EOD1PM) in human sera and specific reactivities. (**a**) Reactivity of anti-sEOD1PM immunoglobulin (Ig) G, IgM, and IgA antibody in human sera. An ELISA plate was coated with solubilized EOD1PM (sEOD1PM) and blocked. Sera were diluted, and the amount of plate-bound Ig was determined using the detection antibody; (**b**) Specificity of the anti- sEOD1PM IgG antibody in human sera. An ELISA plate was coated with sEOD1PM and blocked. Human sera were mixed with serially diluted competitive soluble antigen and then applied to the ELISA plate. The amount of plate-bound Ig was determined using the detection antibody. AgHWE: Polysaccharide fractions from *Agaricus brasiliensis*; ASBG: solubilized cell wall glucan from *Aspergillus niger*.

To further examine anti-paramylon antibody in a more quantifiable and simple way, sEOD1PM was solidified onto ELISA plates, treated with different dilutions of pooled human sera, and the reactivities of detection antibodies of different classes (IgG, IgM, IgA) were analyzed. Anti-EOD1PM antibodies of each class, IgG, IgM, and IgA, were detected, and their binding was concentration-dependent (Figure 4a). Antibody titer analysis revealed that IgG showed the highest titer and reactivity, followed by IgA and IgM. Next, solubilized antigen-based competitive ELISA was used to examine the binding specificity of IgG. sEOD1PM addition suppressed the binding of the solidified sEOD1PM, indicating the presence of EOD1PM-specific IgG in human sera (Figure 4b).

**Figure 5:**
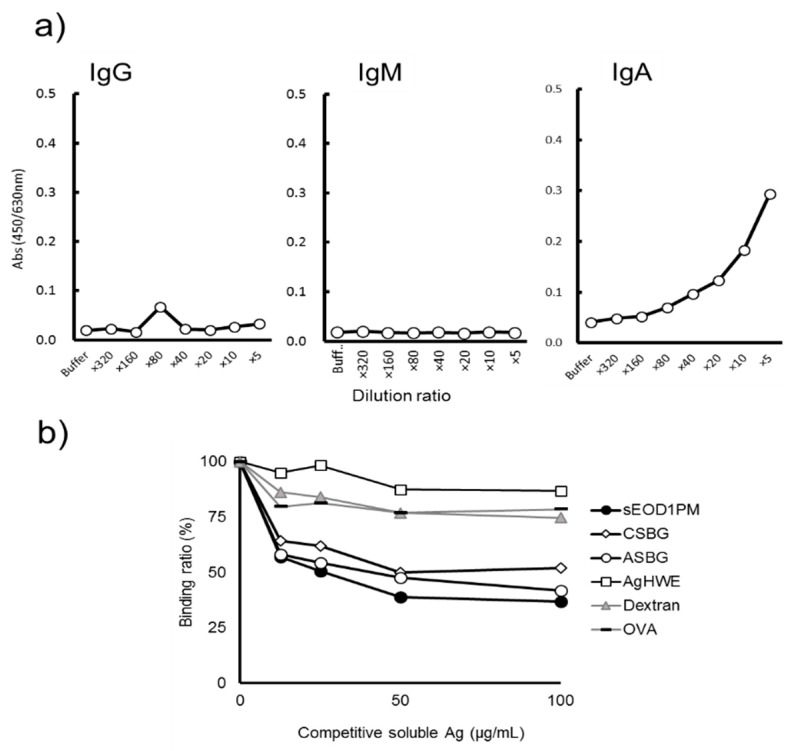
Reactivity of antibody against *Euglena gracilis* EOD-1 paramylon (EOD1PM) in human saliva. (**a**) Reactivity of human salivary anti-sEOD1PM IgG, IgM, and IgA antibody. An ELISA plate was coated with sEOD1PM and blocked. Saliva samples were diluted, and the amount of plate-bound Ig was determined using the detection antibody. (**b**) Specificity of the human salivary anti-sEOD1PM IgA antibody. An ELISA plate was coated with sEOD1PM and blocked. Human saliva was mixed with serially diluted competitive soluble antigen and then applied to the ELISA plate. The amount of plate-bound Ig was determined using the detection antibody.

**Figure 6:**
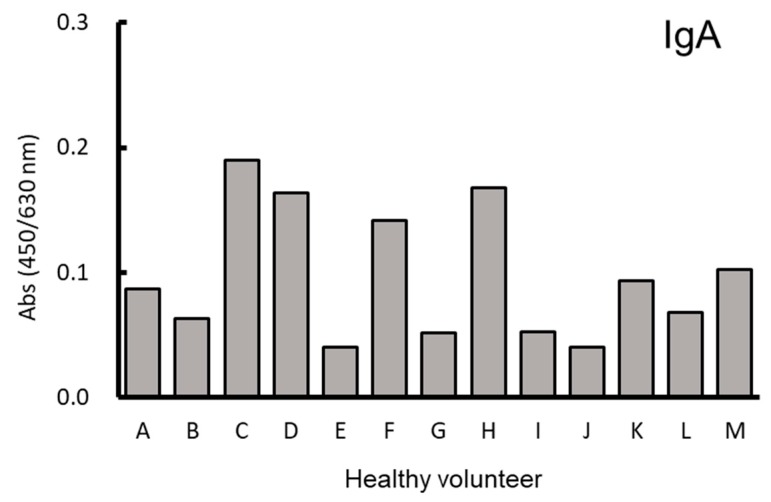
Reactivity of antibody against *Euglena gracilis* EOD-1 paramylon (EOD1PM) in saliva of healthy human volunteers. An ELISA plate was coated with sEOD1PM and blocked. Human saliva samples (*n* = 13) were diluted, and the amount of plate-bound Ig was determined using peroxidase-conjugated anti-human IgA antibody. Each column represents data from one individual.

Next, to further investigate the binding of different classes of salivary anti-EOD1PM antibodies, a dilution series of pooled human saliva was created against sEOD1PM and detection antibodies against the different antibody classes were used to determine the titer values. The IgA antibodies showed a concentration-dependent reactivity, but no reactivity was observed for IgG and IgM antibodies (Figure 5a). Solubilized antigen-based competitive ELISA for salivary antibodies revealed that addition of dextran (α-1, 6-glucan), AgHWE (primarily comprised of β-1, 6-glucan), and OVA did not suppress anti-EOD1PM antibody binding (Figure 5b). However, the addition of sEOD1PM, CSBG, and ASBG did suppress antibody binding. These results indicate that the salivary anti-EOD1PM antibody is specific for β-1, 3-glucan. In addition, the anti-EOD1PM antibody titers in the saliva collected from 13 healthy human volunteers were analyzed. Anti-EOD1PM IgA was detected in the saliva of each healthy volunteer, and different titer values were observed for each volunteer (Figure 6).

### 3.3. Influence of EOD1BM Ingestion on Salivary Anti-EOD1PM IgA Antibody Titer

The concentration of secretory IgA (s-IgA) in the saliva and the secretion rate were determined in study subjects after EOD1BM ingestion. An increase in the concentration and secretion rate of salivary s-IgA was observed in subjects who ingested EOD1BM (Table 1).

**Figure 7:**
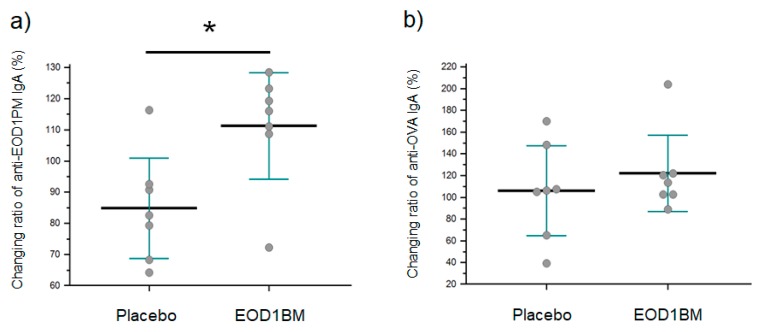
Changes in anti-*Euglena gracilis* EOD-1 paramylon (EOD1PM) antibody titer after EOD1BM ingestion. The anti-EOD1PM antibody titer was measured by ELISA using sEOD1PM-coated plates (*n* = 7). The rate of increase in the titer was calculated using the following formula: Increase in anti-EOD1PM antibody titer (%) = ((anti-EOD1PM antibody titer after ingestion—anti-EOD1PM antibody titer before ingestion)/anti-EOD1PM antibody titer before ingestion) × 100. *: *p* < 0.05 (Mann–Whitney U test, *n* = 7). (**a**) Changes in anti-EOD1PM antibody titer after EOD1BM ingestion. (**b**) Changes in anti-OVA antibody titer after EOD1BM ingestion.

Next, we performed sEOD1PM-based ELISA to determine titer changes for EOD1PM-specific salivary IgA after EOD1BM ingestion. The salivary anti-EOD1PM IgA antibody titer in the EOD1BM Ingestion group was higher than that in the Placebo group (Figure 7). When OVA was used as the contrasting antigen, no significant changes were observed in antibody titers before and after ingestion. These findings indicate that EOD1BM ingestion triggers an EOD1PM-specific immune response in the mucosal surface.

### 3.4. Effect of EOD1BM Ingestion on Health-Related QOL

**Figure 8:**
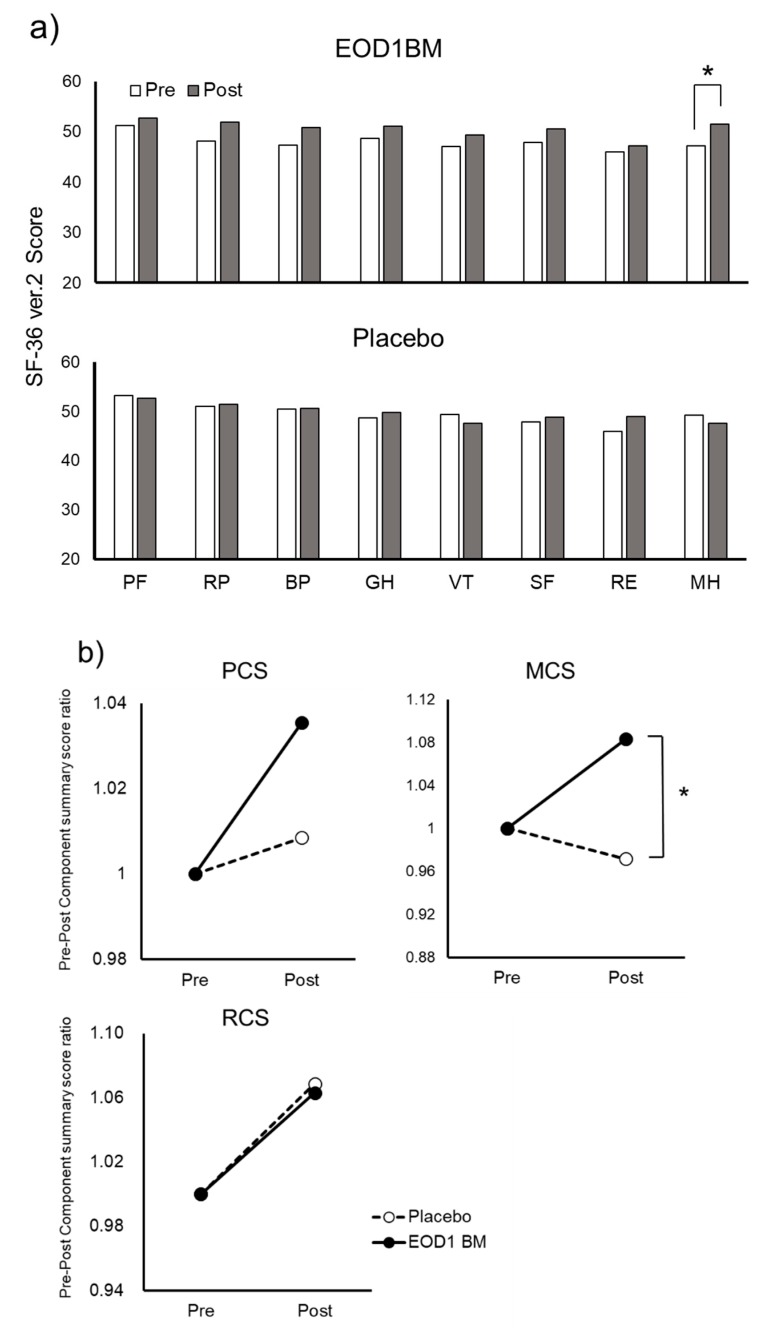
Effect of ingestion of *Euglena gracilis* EOD-1 biomass (EOD1BM) on health-related quality of life (QOL). (**a**) Influence of EOD1BM ingestion on the scores of the subscales of Medical Outcomes Study 36-Item Short Form version 2 (SF-36v2) (*n* = 7). A questionnaire survey from SF-36v2 was administered to the subjects after approximately 4 weeks of EOD1BM ingestion (*n* = 7). The scores before and after EOD1BM ingestion were analyzed and compared. *: *p* < 0.05, Wilcoxon signed-rank test (*n* = 7). (**b**) Effect of EOD1BM ingestion on SF-36v2 component summary score (*n* = 7). The component summary scores of Physical Component Summary (PCS), Mental Component Summary (MCS), and Role–Social Component Summary (RCS) based on the subscale scores before and after EOD1BM ingestion obtained by the administration of the SF-36v2 questionnaire were calculated. The rate of change before and after EOD1BM ingestion was calculated by the following formula: Pre–Post Component summary score ratio = Component summary score after ingestion/Component summary score ratio before ingestion. The changes before and after the ingestion within the group and the differences between the two groups were compared. *: *p* < 0.05, Mann–Whitney U test comparing the placebo and EOD1BM groups at the pre–post ratio (*n* = 7).

To study the effect of EOD1BM ingestion on health-related QOL in all the analyzed volunteers (*n* = 7), we used SF-36v2, a scientific scale with verified reliability and validity. In the EOD1BM ingestion Group, the scores for all the eight subscales, PF, RP, BP, GH, VT, SF, RE, and MH, increased after EOD1BM ingestion (Figure 8). In particular, a statistically significant increase was observed in the mental health category (Figure 8a), and considerable increase was observed in the component summary score for both physical and mental health categories (Figure 8b). However, in the Placebo group, no significant increase was observed in any of the eight subscales. Furthermore, the post-ingestion mental health component summary score in the EOD1BM Ingestion group was significantly higher than that in the Placebo group. In the Role–Social Component Summary Score, there was no difference between the EOD1BM Ingestion and Placebo groups.

## 4. Discussion

In this study, we aimed to investigate the effects of *E. gracilis* EOD1BM on the immunomodulation and health-related QOL in humans. We demonstrate the presence of antibodies against paramylon, a major component of *E. gracilis*, in human serum and the saliva. Paramylon may be involved in inducing an immunomodulatory effect. Furthermore, we demonstrated the applicability of solubilized paramylon-based ELISA for testing and measuring the antibody titers against paramylon. In the EOD1BM Ingestion group, the anti-EOD1PM titer increased. Furthermore, EOD1BM ingestion increased the health-related QOL.

Although several studies have reported the effect of paramylon ingestion in animal models, very few studies investigated the effects of ingestion in humans. Therefore, in this study, we investigated the effect of EOD1BM ingestion, with respect to antibody response, in healthy volunteers. EOD1BM ingestion increased the amount of salivary s-IgA secretion. EOD1BM was able to activate the mucosal immune system. Furthermore, this increase in salivary anti-EOD1PM antibody suggests the induction of an EOD1PM-specific immune response. The ingestion amount of EOD1BM was 500 mg/day and would not cause any problems as a microalgae or dietary fiber [25]. The amount of paramylon was considered to be 350 mg/day. A previous study reported that yeast glucan supplementation of 250 mg/day reduces upper respiratory tract infection after exercise stress [26]; therefore, it was considered normal and effective. Dectin-1, a β-glucan receptor, was reported to be expressed in the intestinal epithelial cells [27]. These findings indicate that EOD1PM induces an immune response via a specific recognition mechanism.

The *E. gracilis* EOD1 strain is known for a high production of the β-glucan paramylon under certain conditions. Various studies have reported the biological activity of β-glucans [28,29,30]. We previously investigated anti-β-glucan antibodies and reported the presence of IgG, IgA, and IgM class antibodies specific to β-1, 3-glucan and β-1, 6-glucan in human sera [13,31]. The present study indicates the presence of an antibody response against EOD1PM, and that the antibody is specific to β-1, 3-glucan. Additionally, the anti-EOD1PM antibody also responded to *Candida* and *Aspergillus*-derived β-glucan. We previously reported that anti-β-glucan antibodies in human sera are generated in response to pathogenic fungi and that these antibodies enhance the antifungal activity of human macrophages [11]. Therefore, anti-EOD1PM antibody would enhance the antifungal activity of macrophages. *Candida* is commonly present in the oral and vaginal cavities and can cause infections on the mucosal surface, whereas *Aspergillus* causes bronchial and pulmonary infections. It is considered that anti-β-glucan IgA, including anti-EOD1PM IgA, which is secreted on the mucosal surface, is also involved in the defense against infectious agents such as pathogenic fungi. Therefore, the increase in anti-EOD1PM antibody titer after EOD1BM ingestion suggests that EOD1BM ingestion could potentially enhance the immune response against such microbial infections.

We used SF-36v2 to examine the influence of EOD1BM ingestion on the health-related QOL of the study subjects. We found that EOD1BM ingestion promoted mental and physical health. Previous studies have indicated that β-glucan ingestion provides a baseline increase in the overall QOL and an increase in QOL for allergy patients [32,33]. Thus, the paramylon contained in EOD1BM may be a contributing factor in this effect. Furthermore, β-glucan ingestion was reported to affect the intestinal flora, especially the proportion of Bacteroides [34,35]. The β-glucan ingestion has been reported to cause changes in microflora by the production of antimicrobial peptides via Dectin-1 [34]. This may also be the case with EOD1BM ingestion. Recent studies have reported a brain-gut connection in which the intestinal flora affect the central nervous system via neuroendocrine, enteric, and immune systems [36]. It was reported that the proportion of *Bacteroides fragilis* is lower in a mouse model with some features of autism [37]. With the progress in studies regarding the relation between the intestinal flora and the mind via the brain–gut connection, it is suggested that the effects of β-glucan ingestion are mediated via such mechanisms.

In the present study, we demonstrate that EOD1BM ingestion for 4 weeks in healthy 30–70-year-old volunteers increased the health-related QOL. Furthermore, EOD1BM ingestion increased the production of an antibody with antigen-specific response to paramylon, the major component of EOD1BM, and was effective in mucosal surface IgA production. These findings suggest that EOD1BM activates the immune response in the mucosal surface. Thus, we report a novel effect of *E. gracilis* EOD-1 in healthy volunteers, although further studies are warranted to elucidate the underlying mechanism. The sample size was chosen based on previous studies of β-glucans in humans. The subjects who were unable to take the measurements at the appropriate time were excluded because the appropriate comparisons could not be performed. The EOD1 BM Ingestion group showed a statistically significant effect compared with the Placebo group. However, because the number and diversity of subjects were limited, further studies with a larger number of more diverse subjects will also be necessary. EOD1BM ingestion resulted in immunomodulation and increased health-related QOL. Thus, we suggest that EOD1BM would be a useful functional food for humans.

## Figures and Tables

**Table 1:** Secretory immunoglobulin A (s-IgA) concentration and secretion rate in saliva before and after the ingestion of *Euglena gracilis* EOD-1 biomass (EOD1BM).

		Pre	Post	Pre-Post Ratio	
s-IgA concentration (μg/mL)	Placebo	31.1 ± 10.7	22.9 ± 7.6	0.77 ± 0.82	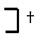
	EOD1BM	23.1 ± 10.5	27.0 ± 4.2	1.41 ± 0.26	
s-IgA secretion rate (μg/min)	Placebo	48.6 ± 11.1	41.6 ± 13.5	0.88 ± 0.28	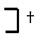
	EOD1BM	34.2 ± 16.5	50.9 ± 13.0 *	1.91 ± 1.29	

All values are shown as mean ± SD (*n* = 7). s-IgA: Secretory immunoglobulin A. * *p* < 0.05, Wilcoxon signed-rank test comparing the results before and after ingestion (*n* = 7). † *p* < 0.05, Mann–Whitney’s U test comparing the pre–post ratios of the placebo and EOD1BM groups (*n* = 7).
